# Patient-derived cells modeling pediatric glioma

**DOI:** 10.18632/aging.101237

**Published:** 2017-05-08

**Authors:** Anna Wenger, Susanna Larsson, Helena Carén

**Affiliations:** Sahlgrenska Cancer Center, Department of Pathology, Institute of Biomedicine, Sahlgrenska Academy, University of Gothenburg, Sweden

**Keywords:** DNA methylation, pediatric, glioblastoma, cancer stem cells, immunodeficient mice

Brain tumors are one of the most common tumors of childhood and there are no effective treatments for high-grade gliomas. One driving force behind glioma is believed to be the cancer stem cells which are thought to be more resistant to treatment and enable relapse, even after an initial anti-tumor response [1, 2]. Research on pediatric brain tumors has been hindered by the lack of relevant model systems. The majority of the very few available cell lines are grown with serum which induces alterations to the cells, making their relevance as a model system questionable [3, 4]. We recently published a study describing patient-derived *in vitro* stem cell cultures that we have established from high-grade glioma [5]. We showed that the established tumor cells could be cultured adherently and in sphere culture, were positive for stem cell markers and responded to differentiation cues. When injected orthotopically into zebrafish and mice the cells initiated tumors resembling the growth patterns of human glioblastoma and we detected a tight span in the survival time within each culture indicating a homogenous culture *in vivo*. These tumor-initiating features together with the expression of stem cell markers and the ability to respond to differentiation cues indicate that the cells are cancer stem cells. We also showed that these cancer stem cells largely retained the chromosomal, mutational and DNA methylation profiles of the tumor samples from which they were generated thus constituting an accurate model system for pediatric brain tumors.

The importance of characterizing primary cell lines over several passages is neglected in many studies which is surprising as it has been shown that alterations occur in cell culture [6], and cells of higher passage will likely be used in experiments as the stock of low-passage cells is limited. It is therefore crucial to establish if changes occur over time and identify which they are to verify the validity of the cells as a model system also at higher passages. Erroneous conclusions might otherwise be drawn from experiments where observed alterations are assumed to be resulting from the investigated treatment but could in fact be a result of high passage. In our study [5] we therefore performed thorough characterization of the cells at three passages; low (passage, p, 5-12), medium (p15-16) and high (p29-30), to confirm the stability of the cells during long-term culture and document any alterations that could be occurring. We observed that the morphology and proliferation rate was largely unaltered between low and high passage but we noticed that some chromosomal alterations occurred at higher passage. Further, the methylation pattern was stable although a few alterations occurred over time with higher passage. However, less than 3% of the investigated sites reversed methylation state (i.e went from methylated to unmethylated or vice versa) at passage 30 and very few of these sites were shared between the cell lines indicating that the changes were not induced by the culture conditions but occurred randomly.

**Figure 1:**
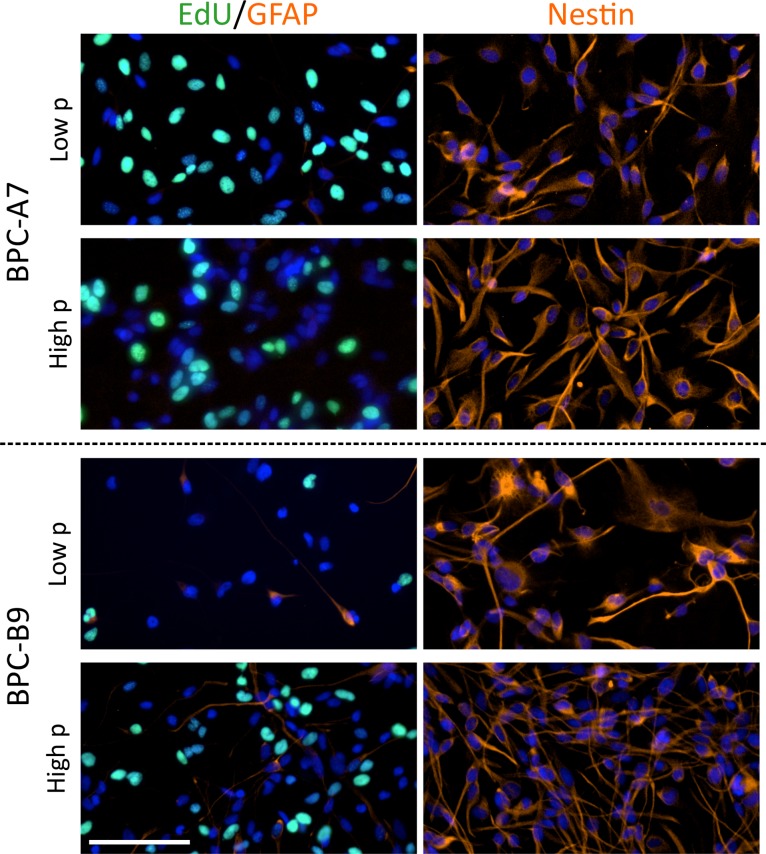
The pediatric tumor cells retain stem cell markers and are proliferative in higher passage The cell lines BPC-A7 and BPC-B9 are proliferative (EdU labeling) also in high passage (p) and have similar expression of the astrocyte marker GFAP in low and higher passage. The stem cell marker nestin is expressed in low passage and retained in high passage demonstrating that the cells are stable in culture. The scale bar is 100 µm.

We have now also examined the expression of stem cell markers in high passage and find that the cells retain stem cell markers such as nestin and are proliferative, as seen by EdU incorporation, (Figure 1) further pointing to the stability of our culture system which preserves the stemness of the cancer stem cells during culture and the cells share features with the tumors they originated from. We have thus presented the first relevant, well-characterized model system that can be used for studying cancer stem cells from pediatric brain tumors which will contribute to increased knowledge on tumor initiation, progression and potentially identify therapeutic targets.
